# Hypoxia-responsive supramolecular systems based on macrocycles and self-assembly structures

**DOI:** 10.4103/mgr.MEDGASRES-D-25-00145

**Published:** 2026-09-12

**Authors:** Farida Bayanovna Gabdrakhmanova, Ekaterina Sergeevna Churbanova, Svetlana Evgenievna Solovieva, Igor Sergeevich Antipin

**Affiliations:** 1 Arbuzov Institute of Organic and Physical Chemistry, FRC Kazan Scientific Center, Russian Academy of Sciences, Kazan, Russian Federation; 2 Department of Organic and Medical Chemistry, Kazan Federal University, Kazan, Russian Federation

**Keywords:** biovisualization, cancer, cucurbiturils, hypoxia, macrocycles, metacyclophanes, paracyclophanes, self-assembly, sensor, supramolecular chemistry, theranostics

## Abstract

Hypoxia is closely associated with tumor progression, metastasis, therapeutic resistance, and diagnostic challenges. The timely detection of hypoxia in live cancer cells is critical for early cancer diagnosis and for monitoring the effectiveness of anticancer therapies. Recently, there has been growing interest in non-covalent supramolecular design strategies for the development of hypoxia-sensitive sensors and prodrugs. Unlike the widely employed covalent approach, supramolecular chemistry eliminates the need for complex and time-consuming synthetic processes. Instead, the individual components of the sensor autonomously self-assemble into the desired structure. This review provides an overview of hypoxia-targeted and hypoxia-activated supramolecular probes, along with their mechanisms of action in tumor diagnosis and therapy. The supramolecular systems considered are classified according to the use of certain classes of macrocycles. The role of macrocyclic derivatives in the various proposed approaches and the response patterns of hypoxic targets will also be analyzed. Various representatives of the macrocyclic platforms, namely cyclodextrins, cucurbiturils, pillararenes, (thia)calixarenes, due to their properties such as molecular recognition, are used as molecular containers-carriers for pharmaceuticals and bio-organic fluorescent dyes. As a result of complex formation, the photostability and the signal-to-noise ratio of dyes increase. In addition, the solubility of hydrophobic guests in water improves, cytotoxicity is reduced, and targeted delivery promotes the accumulation of drugs/dyes in tumor cells.

## Introduction

Hypoxia refers to a condition in which the body or a specific region of the body experiences insufficient oxygen supply at the tissue level.^1^,^2^ The term “normoxia” describes the partial pressure of oxygen in the inspired air, typically between 10–21% oxygen (O_2_), whereas hypoxia is characterized by low oxygen tension, generally in the range of 1–5% oxygen. Within most organs, the physiological oxygen level of tissues is hypoxic. For example, arterial blood oxygen is approximately 13%, and oxygen concentrations within tissues decrease as the distance from blood vessels increases. Oxygen tension varies across different tissues: from 0.5% to 7% in the brain; 4–12% in the lung, fat, skin, bone, and cartilage.^3^ The phenomenon of hypoxia is associated with the development of various pathological conditions and, notably, serves as a key indicator of tumor aggressiveness. Tumor growth induces changes in the vascular system and blood flow, leading to the emergence of regions with oxygen gradients and areas of acute hypoxia (less than 1.4% oxygen) in rapidly growing tumors.^4^,^5^,^6^,^7^,^8^ Hypoxia is a key factor in tumor formation, growth and resistance to therapy,^9^ particularly chemotherapy, radiation therapy and photodynamic therapy (PDT).^4^ Hypoxia poses a significant challenge in cancer treatment. Radiation therapy, a primary modality in cancer treatment, demonstrates substantially reduced efficacy in hypoxic conditions.^10^ Cancer cells in hypoxic environments are 2–3 times more resistant to ionizing radiation compared to normoxic cells.^11^ One proposed explanation for this resistance is the inadequate or insufficient delivery of therapeutic agents to hypoxic regions in large tumors.^9^ Therefore, the timely identification of hypoxic regions is crucial for the effective diagnosis and treatment of cancer. As a result, there is considerable interest in the development of methods for visualizing hypoxia.

The hypoxic environment surrounding tumors often leads to the formation of a localized microenvironment characterized by relatively low pH, variable oxygen concentration, and altered levels of hypoxia-inducible factor 1-alpha, and carbonic anhydrase-IX. The reduced oxygen content in hypoxic cells results in increased reductive stress,^12^ thereby triggering more rapid biological repair processes, which in turn cause the overexpression of various classes of intracellular enzymes such as nitroreductase, dihydronicotinamide adenine dinucleotide phosphate dehydrogenase, azoreductase, cytochrome P450, and quinoneoxidoreductase.^13^,^14^ The determination of the location and expression levels of hypoxia-related enzymes in living cancer cells using hypoxia-activated molecular targeting techniques is crucial for early cancer diagnosis and the monitoring of the efficacy of anticancer therapies.^15^,^16^ Thus, the unique biological characteristics of the microenvironment in hypoxic cancer tissues render them a specific target for hypoxia-sensitive systems. These findings have led researchers to design and develop hypoxia-activated prodrugs, which can be activated by these enzymes for the targeted therapy of hypoxic tumors (**Figure 1**).^17^,^18^,^19^

**Figure 1:**
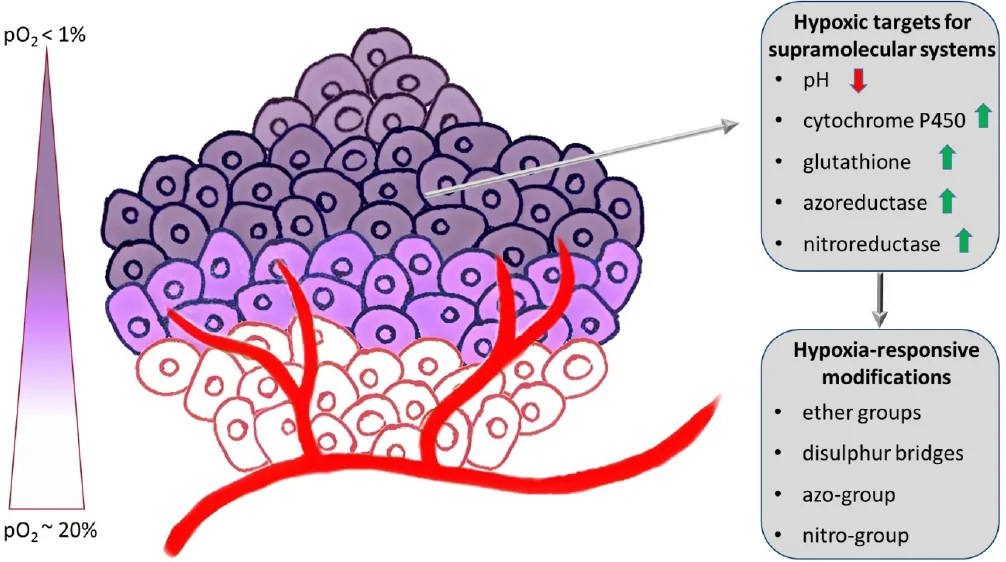
Features of the hypoxic microenvironment in cancer tissues as targets for supramolecular hypoxia-responsive systems. Created with Microsoft PowerPoint 2013. pO_2_: Oxygen partial pressure.

A variety of invasive and non-invasive approaches have been developed to measure tumor oxygenation, including oxygen-sensitive electrodes and hypoxia marker techniques using various labels that can be detected by different methods, such as positron emission tomography,^20^ single photon emission computed tomography,^21^ magnetic resonance imaging,^22^ photoacoustic imaging,^23^ and fluorescence imaging.^24^ Nowadays, specialized fluorescent probes are more commonly employed. The advantages of fluorescent probes over radioactive ones include lower cost, the ability to be stored indefinitely, and the absence of ionizing radiation exposure for both the researcher and the participant. However, the ability of visible light to penetrate tissue limits its use to certain preclinical and clinical contexts.^25^,^26^,^27^ Most published research focuses on the generation of covalently linked probes, which are molecules connected by strong covalent bonds, one of which serves as a hypoxia-sensing motif while the other serves as a sensor or recognition component. This “covalent approach” is discussed extensively in excellent reviews and monographs.^28^,^29^ Despite their effectiveness in creating hypoxia-sensitive systems, these sensors have certain drawbacks, including complex molecular design, multi-step synthesis, and high cost.^30^,^31^,^32^

More recently, a “supramolecular approach” has emerged, based on weak, multiple interactions such as hydrogen bonds, van der Waals forces, coordination bonds, and guest–host interactions between molecules. Properties such as self-organization, adaptability, and high selectivity allow these probes to be more sensitive to specific molecules and respond to characteristic markers associated with hypoxia. One of the main advantages of this approach is the ability of self-assembly, eliminating the need for lengthy and complex syntheses. Instead, the components of the sensor spontaneously organize into the desired structure. Supramolecular materials responsive to external factors activated by hypoxic microenvironments are widely employed in biomedical fields for drug delivery, diagnostics, and image-guided treatments.

The formation of host–guest complexes, which involves the specific recognition of host and guest molecules, is a widely employed strategy in the development of supramolecular sensors.^32^,^33^ Macrocyclic molecules such as cyclodextrins,^34^ calixarenes,^35^ cucurbiturils,^36^ and pillararenes^37^ demonstrate exceptional host and guest capabilities due to their unique size-variable cavities.

In the review, we consider supramolecular systems and their strategies of action, categorized according to the use of specific classes of macrocycles. The role of macrocyclic derivatives in different proposed approaches and the response patterns of hypoxic targets will also be analyzed.

## Search Strategy

The literature search was carried out in Google Scholar and SciFinder-n using keywords, such as hypoxia, cancer, supramolecular chemistry, biovisualization, macrocycles, metacyclophanes, paracyclophanes, cucurbiturils, self-assembly, sensor, and theranostics. The main criterion for selection was the use of a non-covalent method to obtain hypoxia-responsive systems.

## Sensors and Prodrugs Based on (Thia)calix[n]arenes

Calix[n]arenes are macrocyclic compounds consisting of phenolic units connected by methylene groups at the 2,6-positions, with defined upper and lower rims and a central ring.^38^ In their analogues, thiacalixarenes, the methylene groups are replaced by sulfur atoms.^39^ The name of this class of macrocycles derives from their resemblance to a type of Greek vase called a calyx krater.^40^ It is well established that (thia)calix[n]arenes offer several advantages over typical ligands, including the ability to incorporate small organic molecules or fragments of large biomolecules into molecular cavities, leading to the formation of host–guest complexes.^41^,^42^ Additionally, (thia)calix[n]arenes can be readily functionalized with a variety of receptor groups.^43^,^44^ The incorporation of groups into a single molecule and the fixing of their specific, predetermined spatial arrangement allow for a variety of possibilities in functionalization, both on the upper and lower rims with receptor groups of different natures.^45^ Moreover, the low toxicity of calixarenes further enhances the potential of these molecules for biomedical applications.^46^,^47^,^48^,^49^,^50^,^51^ It was therefore proposed that supramolecular complexes with fluorescent dyes based on macrocyclic azo derivatives of (thia)calix[4]arene could serve as hypoxia-sensitive sensors.^52^,^53^,^54^,^55^,^56^ The dye is pre-incorporated into the cavity of the azo derivative, forming a host–guest complex. The formation of this complex leads to the quenching of fluorescence. However, in the presence of a hypoxic environment characterized by increased azoreductase content, the azo groups of the macrocycle are reduced to amino groups. This results in the release of the dye and subsequent restoration of the fluorescence intensity (**Figure 2**). The proposed strategy has the potential to be utilized not only in conjunction with dyes but also in the context of therapeutic agents employed in the treatment of cancer.

**Figure 2:**
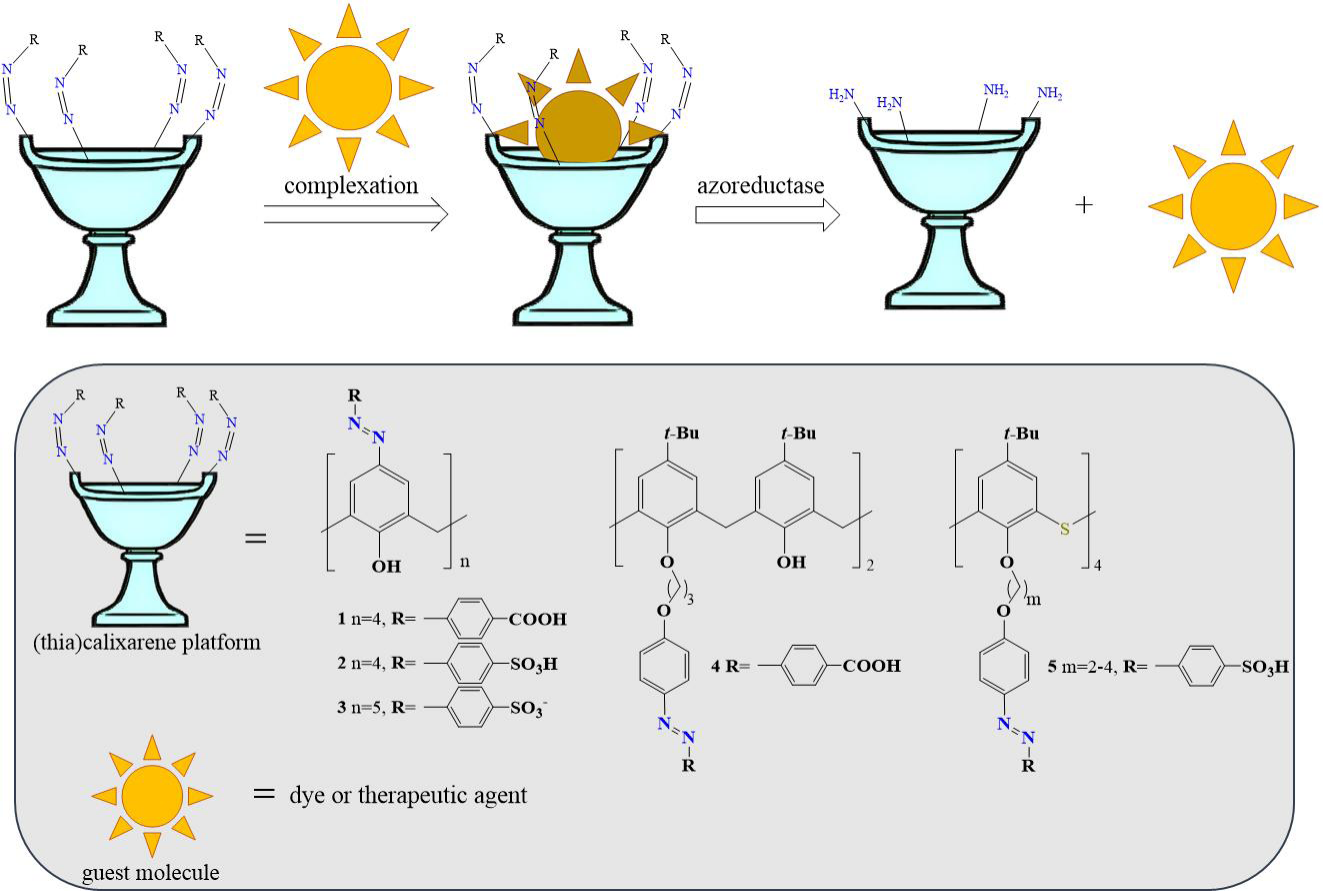
The general idea behind the use of (thia)calixarenes. Initially, a complex forms between the azo derivative (thia)calix[4]arene and the guest molecule. Under the influence of azoreductase, the azo groups are reduced, resulting in the release of the guest molecule. Created with ChemBioDraw Ultra 12.0.

To enhance complexation with cationic rhodamine dyes, azo derivatives of (thia)calix[4]arenes 1-2,4-5 were also functionalized with anionic sulfonate and carboxylate groups on the upper or lower rims (**Figure 2**). A systematic study of the obtained complexes was carried out using ultraviolet-visible and fluorescence spectroscopy as well as nuclear magnetic resonance techniques.^52^,^53^,^54^,^55^,^56^ As a proof-of-concept, the use of the binary system in hypoxia-selective imaging in living cells was investigated. A549 cancer cells were incubated with azocalixarene 1–rhodamine 123 complex in both normoxic and hypoxic conditions. The incubation of A549 cells with the binary system under normoxic conditions resulted in a markedly low fluorescence signal. In contrast, when the cells were cultured under hypoxic conditions, a significantly brighter fluorescence was observed within the cells. These findings demonstrate the efficacy of the azocalixarene 1–rhodamine 123 reporter pair in detecting hypoxia in living cells.^52^

The aforementioned approach was also demonstrated with other guest molecules. Accordingly, a hydrogel based on azocalixarene 2 and hyaluronic acid grafted with the dye N-ethylamino-4-azetidinyl-1,8-naphthalimide was prepared.^57^ The fluorescence signal of the dye in the supramolecular hydrogel is quenched by azocalixarene. Given that azocalixarene exhibits hypoxia-responsive characteristics, the azo bond is reduced under hypoxic conditions. After the azo bond in calixarene is reduced and cleaved, the N-ethylamino-4-azetidinyl-1,8-naphthalimide dye is released. As a result, the fluorescence signal is renewed. Thus, a novel host-responsive supramolecular hydrogel has been developed, capable of monitoring the response to a hypoxic environment through fluorescence recovery.

The further development of the approach utilizing azocalixarene probes for visualizing hypoxia has led to the creation of prodrugs for anticancer therapy. Specifically, the substitution of the dye with a therapeutic agent in azocalixarene 2 complexes has resulted in the proposal of novel hypoxia-responsive drug delivery systems for tumors. For example, a red-emitting cationic aggregation-induced photosensitizer has been employed as a guest molecule in this context.^58^ It is anticipated that this proposed strategy will facilitate the development of efficient image-guided PDT.

The complexation properties of carboxyazocalix[4]arene 1 with a series of chemotherapeutic drugs were also investigated. The present study demonstrated that the complex of calixarene with doxorubicin (DOX) exhibited a significant antitumor effect. In contrast, mice treated with DOX at the same dosage showed insignificant tumor suppression, suggesting that azocalixarene may reduce the effective dose of the cancer drug due to optimized *in vivo* distribution and increased tumor accumulation.^59^ Of particular note is the promising use of the photothermal agent IR780 in a guest role within an azocalixarene 2 complex.^60^ The encapsulation of IR780 within the macrocycle’s cavity effectively upgrades the solubility, photostability, and photothermal conversion issues commonly associated with organic small molecule photothermal agents. This approach also facilitates the development of imaging-guided photothermal therapy (PTT). The supramolecular PTT formulation achieves selective tumor imaging and high therapeutic efficacy, thereby enabling highly effective tumor ablation while minimizing damage to surrounding normal tissues.

Another guest variant in the carboxylic azocalixarene complex is the low molecular weight radiosensitizer banoxantrone dihydrochloride (AQ4N).^61^ AQ4N is recognized as a highly selective bioreductive drug activated in hypoxic tumor cells, where it is primarily toxic. It has been shown that combining AQ4N with radiotherapy or chemotherapy significantly enhances the efficacy of these treatments.^62^ However, AQ4N exhibits salt-like properties that present several challenges, such as excessive aqueous solubility, low cellular uptake, and rapid *in vivo* elimination.^63^ It has been suggested that carboxylic azocalixarene 1 could be employed as a molecular container to deliver and release AQ4N in hypoxic cancer cells. *In vivo* animal experiments have demonstrated that supramolecular radiotherapy achieves greater therapeutic efficacy compared to free radiosensitizers, while also reducing the required drug dosage and radiation dose, thus minimizing the risk of damage to normal tissues.^63^ The enhanced radiotherapeutic efficacy of this strategy can be attributed primarily to the improved tumor accumulation and cellular internalization of the drug, achieved through sophisticated host–guest complexation.

The cyclic depsipeptide BE-43547A2 represents an additional example of a guest molecule that has been observed to form a complex with sulfonated azocalix[5]arene 3.^64^ BE-43547A2 exhibits enhanced cytotoxicity against hypoxic pancreatic cancer cells; however, its poor solubility limits its application.^65^ The encapsulation of BE-43547A2 in sulfonated azocalix[5]arene results in the formation of a hypoxia-responsive supramolecular double complex. This dual complex, which selectively releases the payload within cancer cells, markedly suppresses tumor growth at a low dose in a mouse model of pancreatic cancer cell xenograft without developing systemic toxicity.

Finally, nanoparticles with antitumor properties have been proposed based on azocalixarenes 6 (**Figure 3**).^66^ A supramolecular polymer was prepared based on the dimeric sulfonate modified azocalix[4]arene, which subsequently self-organized into nanoparticles. Camptothecin, an anticancer drug functionalized with a disulfide-containing linker for glutathione sensitivity, was selected as the guest molecule. The unique dual-responsive nanoparticle exhibited exceptional stability against dilution and biological interference, as well as precise camptothecin release under conditions of glutathione and azoreductase activity. *In vitro* and *in vivo* experiments demonstrated that dual-responsive supramolecular polymer into nanoparticles exhibited favorable biosafety and tumor-suppressive effects.

**Figure 3:**
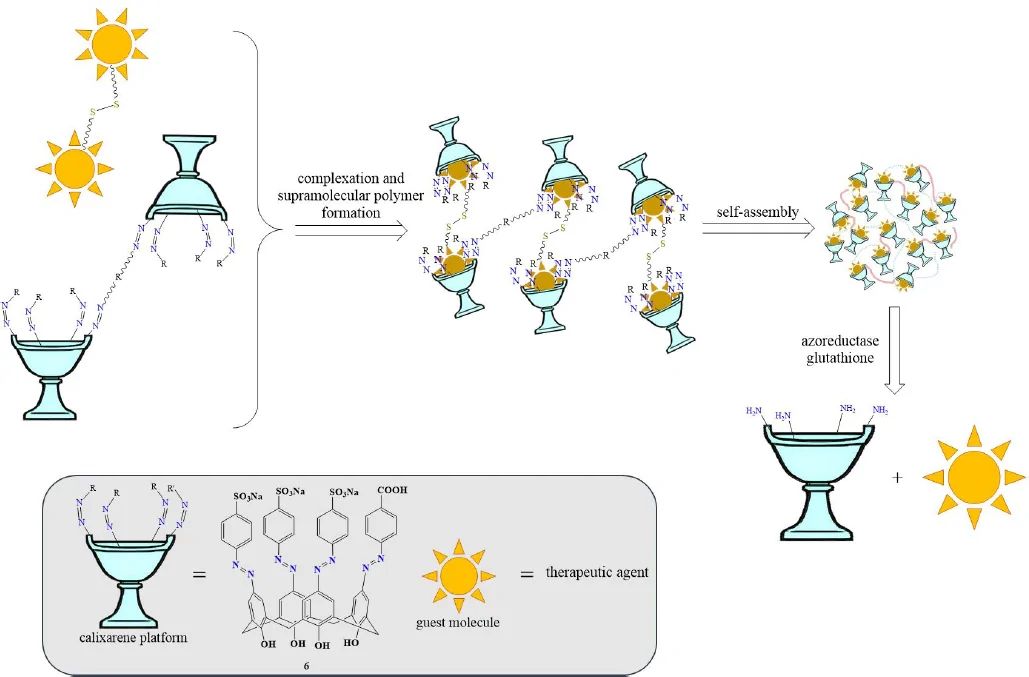
The application strategy of azocalixarene-based nanoparticles. The complex is formed between the dimeric sulfonate-modified azocalix[4]arene and the guest molecule (specifically, the therapeutic agent), leading to the formation of a polymer. Under the influence of azoreductase and glutathione, the azo groups and disulfide linkers are reduced, resulting in the release of the therapeutic agent. Created with ChemBioDraw Ultra 12.0.

Thus, the advancement of supramolecular hypoxia-sensitive systems based on calixarenes is currently focused on azo derivatives that target azoreductase. Various guest molecules in dual systems have been proposed, and cone and 1,3-alternate conformations have been explored. The azo derivatives at the upper rim based of classical calix[4]arene have been the subject of the most comprehensive research.

## Cucurbiturils as Part of Sensory Systems and Prodrugs

Another representative of macrocycles, cucurbiturils, has also been utilized to develop hypoxia-targeted theranostic agents. Cucurbit[n]urils (*n* = 5–8, 10, 14) form a unique family of macrocyclic gourd-shaped host molecules that are well established as excellent ‘intermediates’ for both bioimaging and chemotherapeutics.^67^,^68^,^69^

In the context of hypoxia-sensitive systems, the most frequently proposed complexes involve cucurbituril and AQ4N. As previously mentioned, AQ4N is a prodrug that inhibits topoisomerase II, which is overexpressed in hypoxic cells.^70^ The authors proposed the use of micelles derived from a polyethylene glycol system that were dually tagged with hydrophilic cucurbit[7]uril 7 and hydrophobic Chlorin e6 (Ce6) on each end, for synergistic antitumor therapy via PDT of Ce6 and chemotherapy using a hypoxia-responsive prodrug AQ4N, which was loaded into the cavity of cucurbit[7]uril 7 (**Figure 4**).^71^ The resulting micelles were then subjected to further modular functionalization with folate through a strong host–guest interaction between folate-amantadine and cucurbituril. This nanoplatform demonstrated enhanced cellular uptake, negligible toxicity and good biocompatibility, as well as improved intracellular reactive oxygen species generation efficiency. Of particular significance was the *in vivo* evaluation of the modular nanoplatform, which revealed a synergistic antitumor efficacy between Ce6 PDT and hypoxia-activated chemotherapy AQ4N (which can be converted to chemotherapeutic 1,4-bis[[2-(dimethylaminoethyl]-amino]5,8-dihydroxyanthracene-9,10-dione for tumor chemotherapy in response to the enhanced hypoxic tumor microenvironment during PDT). This study not only provides a novel nanoplatform for synergistic photodynamic-chemotherapeutic therapy but also offers important new insights for the design and development of a multifunctional supramolecular drug delivery system.

**Figure 4:**
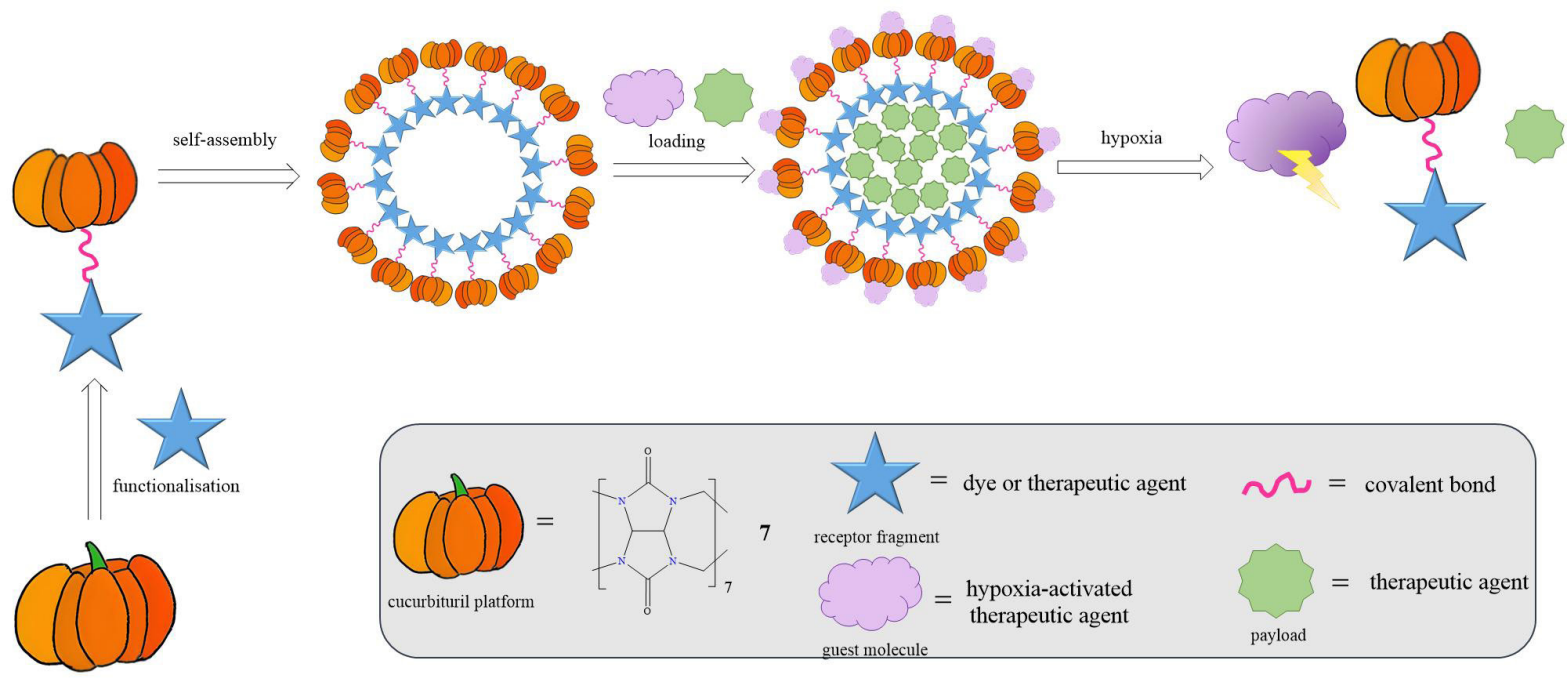
The general strategy for producing hypoxia-targeted cucurbituril-based micelles for anticancer therapy. In the initial stage, the cucurbituril platform is functionalized with receptor fragments. The next step involves the self-assembly of cucurbituril derivatives into nanoaggregates. Subsequently, these nanoaggregates are loaded with therapeutic agents. Under hypoxic conditions, the resulting nanoaggregates break down, leading to the release of the therapeutic agents. Created with ChemBioDraw Ultra 12.0.

Additionally, there are examples of AQ4N complexes where cucurbit[7]uril 7 is modified with a fluorophore, altering its fluorescence in weakly acidic environments, a characteristic feature of hypoxia (**Figure 4**).^72^ It is important to note that the photosensitizer functionalized cucurbituril has the capacity for spontaneous self-organization into nanoaggregates in aqueous solutions. Due to the guest binding properties of cucurbituril, oxaliplatin was incorporated into the obtained nanoaggregates for combined PDT and supramolecular chemotherapy. To demonstrate the modular versatility of this supramolecular system, the hypoxia-activated prodrug AQ4N was loaded into nanoaggregates, which demonstrated synergistic antitumor effects on a multicellular tumor spheroid model. Such systems are advantageous for multi-target theranostic applications, as they permit the simultaneous imaging and treatment of tumors.

Cucurbit[7]uril supramolecular complexes are also employed in PTT.^73^ In this instance, a supramolecular complex composed of two cucurbit[7]uril 7 molecules and one perylenediimide derivative molecule was proposed (**Figure 5**). This complex is capable of generating delocalized radical anions in a reductive hypoxic environment. Thus, a supramolecular agent for targeted PTT was developed. Furthermore, the mRNA expression of hypoxia-inducible factor 1-alpha was demonstrated to return to normal levels following PTT treatment. It is anticipated that this hypoxia-induced PTT will facilitate the development of improved techniques for treating hypoxic tumors with greater specificity and safety.

**Figure 5:**
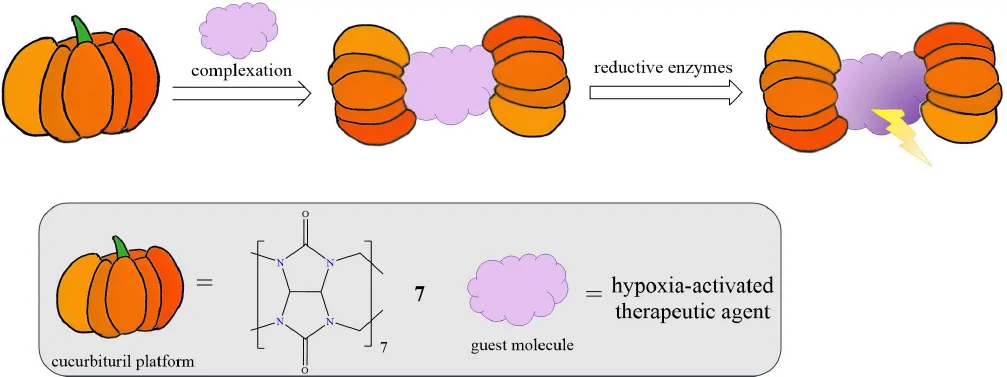
The preparation of supramolecular complexes based on cucurbiturils for anticancer therapy. A complex is formed consisting of two molecules of cucurbit[7]uril and one molecule of a hypoxia-activated therapeutic agent, specifically a perylene diimide derivative. Under hypoxic conditions, the therapeutic agent is activated. Created with ChemBioDraw Ultra 12.0.

Nanoparticles based on poly-cucurbit[7]uril, with multiple binding sites, have also been proposed for the encapsulation of chemotherapeutic drugs, including oxaliplatin and a mitochondria-targeted cytotoxic peptide.^74^ Meanwhile, the inactive competitors with acid-cleavable protection groups on the nanoparticle shell can readily recover under the acidic hypoxic tumor, competitively replacing and releasing the encapsulated combination of drugs (**Figure 6**). This strategy of self-motivated supramolecular combination chemotherapy provides a new method for efficient combination of chemical drugs and bioactive peptides with on-demand drug release, and opens up significant potential for supramolecular chemotherapy in the fight against drug resistance in cancers.

**Figure 6:**
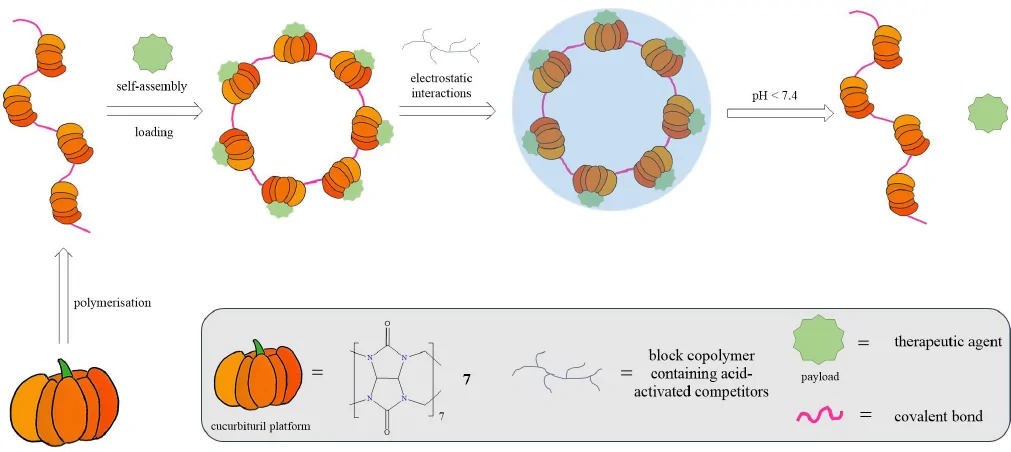
The preparation of nanoparticles based on poly-cucurbit[7]uril for chemotherapy. A polymer based on cucurbit[7]uril is synthesized. Next, nanoparticles loaded with therapeutic agents are formed through the self-assembly of polycucurbituril. In the subsequent step, the nanoparticle shells are treated with acid-cleavable protection groups. In an acidic hypoxic environment, these shells degrade, resulting in the release of the encapsulated drug. Created with ChemBioDraw Ultra 12.0.

An additional illustration of hypoxia-responsive systems involves the formation of covalently self-organizing polymeric nanocapsules based on perhydroxycucurbit[6]uril 8 and a ditopicazobenzene derivative (**Figure 7**). The surface of the resulting nanocapsules can be non-covalently functionalized with payloads. To demonstrate proof of concept, folate-functionalized nanocapsules were used to target the delivery of the payload to cancer cells both *in vitro* and *in vivo*. The release of the payload was observed under the action of azoreductase in a hypoxic environment. It is thus proposed that the aforementioned systems may provide a robust, hypoxia-responsive polymeric platform with a non-covalently customizable surface to targeted drug delivery to meet current medical needs in the treatment of cancer and inflammation, where hypoxia is the dominant pathological hallmark. This work may also stimulate further interest and research into stimulus-responsive macrocycle-based nanocapsules for biomedical applications.^75^

**Figure 7:**
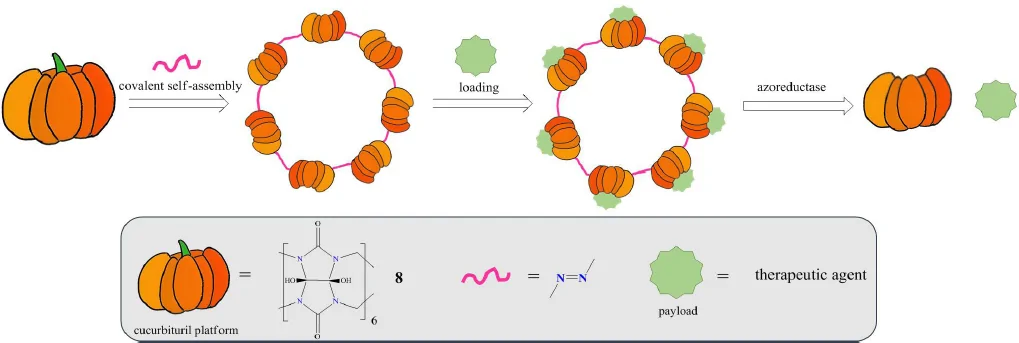
The preparation of cucurbituril-based polymeric nanocapsules for targeted delivery and hypoxia-responsive release. Initially, covalently self-organizing polymeric nanocapsules containing azo groups are synthesized. These nanocapsules are then loaded with therapeutic agents. Under hypoxic conditions, the action of azoreductase leads to the degradation of the nanocapsules, resulting in the release of the encapsulated drug. Created with ChemBioDraw Ultra 12.0.

In conclusion, cucurbiturils generally serve as containers in the presented works, and the targeting of hypoxic cancer cells is achieved either through specific guest molecules or through additional modifications of the nanoparticles. Furthermore, the potential for visualizing hypoxia in the context of cucurbiturils remains underexplored.

## Cyclodextrins in Nanoaggregates for Imaging and Therapy of Hypoxia

The next class of macrocyclic molecules widely employed in the pharmaceutical industry is cyclodextrins. This is due to the fact that they are biocompatible and biodegradable.^76^ Cyclodextrins consist of 6 to 12 (α-1,4)-linked α-D-glucopyranose units. Due to the specific conformation of the glucopyranose units in the chain, they exhibit a truncated cone shape, with a slightly hydrophobic central cavity and a hydrophilic exterior.^77^

Cyclodextrins are commonly used in the construction of hypoxia-sensitive sensors and prodrugs. A non-covalent hypoxia-responsive turn-on fluorescence probe has been developed using sulfato-β-cyclodextrin 9, an azobenzene derivative, and fluorochrome rhodamine 123 (**Figure 8**).^78^ The aggregation process is driven by the electrostatic interaction between the negatively charged sulfato-β-cyclodextrin 9 and the positively charged rhodamine. During the process of supramolecular assembly, the fluorescence of the rhodamine dye is quenched. However, under hypoxic conditions, the azo-bond in azobenzene is reduced, leading to the disruption of the nanoparticle and the release of the dye. This model was validated through successful testing on A549 cells.

**Figure 8:**
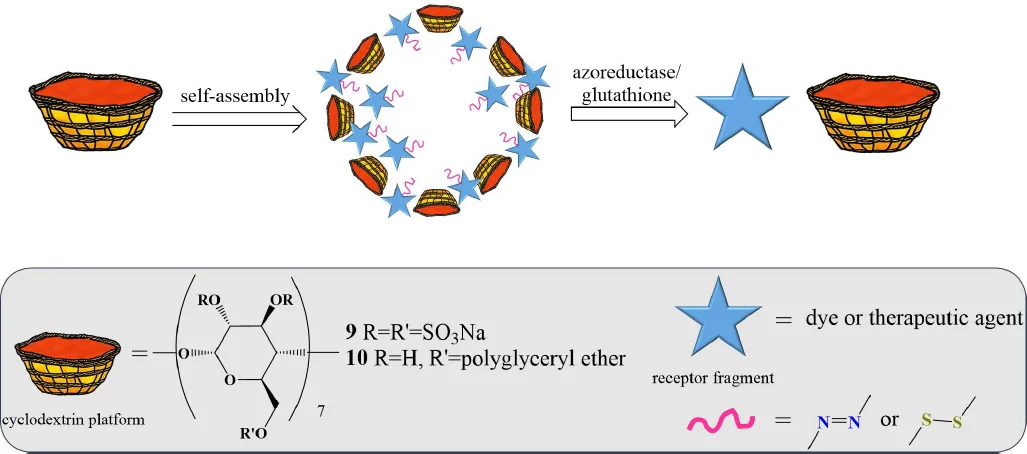
The utilization of cyclodextrins in the self-assembly of hypoxia-sensing systems. Nanoparticles based on cyclodextrin and a dye or therapeutic agent containing azo linkages are synthesized. Under hypoxic conditions, the nanoparticles are degraded by azoreductase, resulting in the release of the dye or therapeutic agent. Created with ChemBioDraw Ultra 12.0.

A cyclodextrin-based theranostic agent for image-guided PDT has also been developed.^79^ The modular theranostic agent is composed of a benzimidazole-modified polyporphyrin core rich in disulfide bonds, a shell consisting of a hyperbranched glycidyl ester of cyclodextrin 10 modified with folic acid, and a hypoxia-activated fluorescent probe (**Figure 8**). Supramolecular self-assembly of the host–guest benzimidazole and cyclodextrin leads to the formation of a hierarchical core-shell structure that can be deconstructed in a weakly acidic tumor microenvironment. In the hypoxic tumor environment, the photodynamic module is activated by the action of glutathione and the fluorescence of the indicator module is “switched on” under the influence of nitroreductase. This allows hypoxia to be visualized. Thus, the proposed supramolecular system may be useful for surgical resection under the control of fluorescence imaging of hypoxia, and PDT against heterogeneous tumors.

Deng et al.^80^ proposed the use of supramolecular nanocarriers for enhanced PDT based on an α-cyclodextrin 11 (**Figure 9**). The combined therapeutic effect is achieved by integrating a glutathione-sensitive nitric oxide prodrug, as well as an S-nitrosothiol and light-activated Ce6 into a macrocyclic platform. It is evident that laser-irradiated supramolecular systems have the potential to significantly inhibit cancer cell proliferation and suppress tumor growth through the superior synergistic effect of nitric oxide and PDT. The ability of nitric oxide to relieve hypoxia and deplete intracellular glutathione is of great importance as a general strategy not only for PDT but also for radiotherapy, drug resistance reversal, and other scenarios.

**Figure 9:**
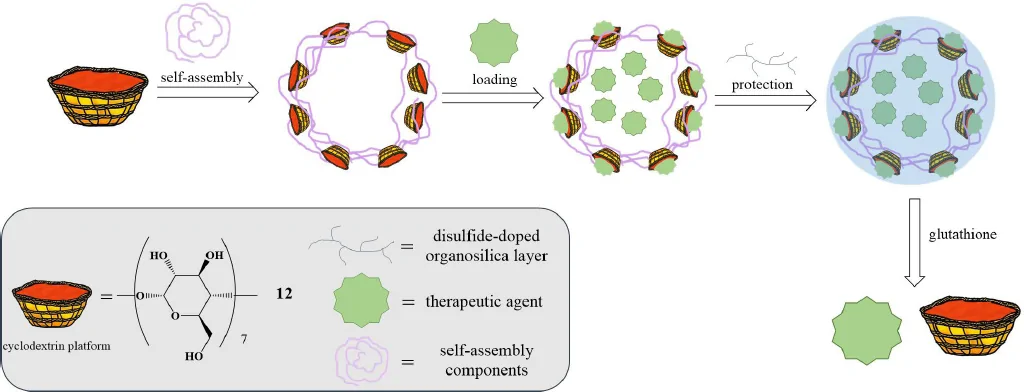
The application of supramolecular nanocarriers for enhanced photodynamic therapy. In the first stage, the cyclodextrin platform is functionalized with hypoxia-activated therapeutic agents. Next, nanoparticles are formed from the resulting cyclodextrin derivatives through a self-assembly process. The therapeutic agents are activated under hypoxic conditions. Created with ChemBioDraw Ultra 12.0.

Additionally, β-cyclodextrin 12-based hybrid vesicles with dual glutathione/pH response were developed for the encapsulation and simultaneous delivery of the biomacromolecule glucose oxidase and the chemotherapeutic drug docetaxel for synergistic enzymatic/chemotherapeutic tumor therapy.^81^ These vesicles are constructed by the self-assembly of β-cyclodextrin-poly(ε-caprolactone), ferrocene (Fc)-poly(acrylic acid) and pillar[5]arenes via terminal β-cyclodextrin/Fc host–guest interaction, followed by cross-linking with 3-mercaptopropyltrimethoxysilane and poly(ethylene glycol) modification. Under the influence of glutathione in the hypoxic tumor microenvironment, primary disassembly/degradation of the disulfide-linked organosilicate layer of the vesicle occurs (**Figure 10**). The release of glucose oxidase leads to a cytotoxic effect and triggers a mechanism of disruption of the guest–host complex through the formation of hydrogen peroxide, leading to the release of docetaxel. In this way, a strategy for synergistic enzymatic and chemo-tumor therapy can be implemented.

**Figure 10:**
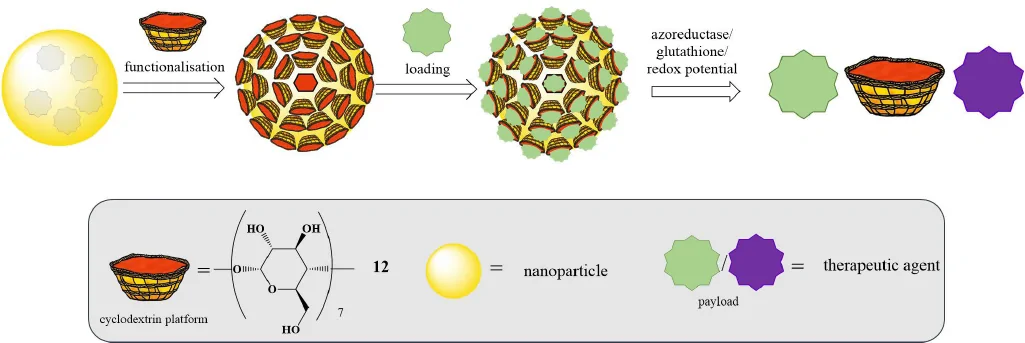
The application of cyclodextrin-based hybrid vesicles for synergistic enzymatic/chemotherapeutic tumor therapy. The synthesis yields nanoparticles based on β-cyclodextrin-poly(ε-caprolactone), ferrocene (Fc)-poly(acrylic acid), and pillar[5]arenes. These nanoparticles are then loaded with therapeutic agents and coated with a disulfide-doped organosilica layer. Under hypoxic conditions, the nanoparticles are degraded by glutathione, resulting in the release of the therapeutic agents. Created with ChemBioDraw Ultra 12.0.

While previous works utilized cyclodextrins in the nanoparticle formation step, the following examples illustrate an alternative strategy: the functionalization of the surface of the prepared nanoparticles for additional useful loading (**Figure 11**). In particular, an amphiphilic polymer based on β-cyclodextrin 12 was anchored on Fc-capped gold nanoparticles via a guest–host interaction.^82^ The initial dissociation of the host–guest interactions and subsequent release of the aurum nanoparticles by cancer-specific reactive oxygen species results in the release of glucose oxidase, which consumes glucose and oxygen, generating hydrogen peroxide and inducing the hypoxic tumor microenvironment. This, in turn, triggers burst payload release and promoter activation, thus allowing for synergistic starvation and gene therapy for cancer. Furthermore, the researchers functionalize the nanoparticle surface with cyclodextrins for subsequent loading with therapeutic agents.^83^ In this instance, the guest molecule is also modified with an azo moiety, which is reconstituted in a hypoxic environment and results in the release of the encapsulated drugs. In another study, disulfide-bridged and DOX-embedded degradable silicon nanoparticles are proposed.^84^ To realize gene and drug co-direction, the nanoparticle surface is readily functionalized with an assembled polycation that includes one β-cyclodextrin core and two ethanolamine-functionalized poly(glycidyl methacrylate) arms. The redox-sensitive self-destruction gives the nanoparticles a better ability to release the anticancer drug DOX, while the low-toxicity hydroxyl-rich cyclodextrin brushes can efficiently deliver genes for cancer therapy. The present study demonstrates that the enhanced antitumor effect is achieved through the integration of gene and chemotherapy treatments. This research provides a foundation for the development of innovative codelivery carriers that exhibit a balance of biodegradability, stimuli-responsiveness, and controlled release properties.

**Figure 11:**
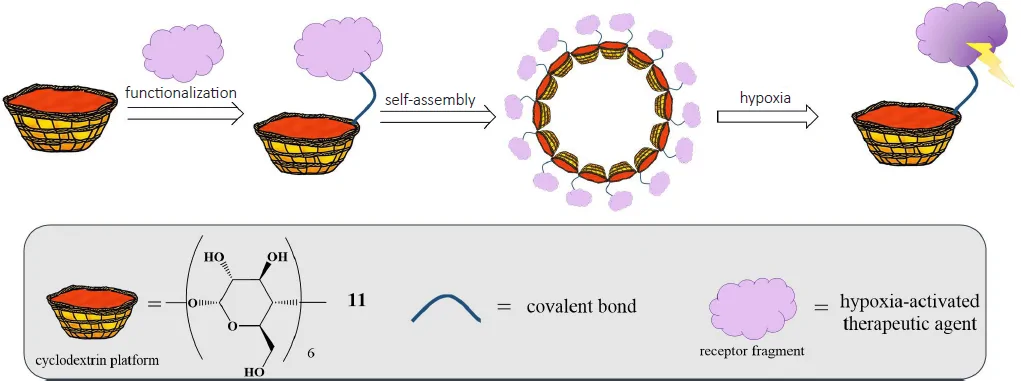
The general strategy of cyclodextrins in the functionalization of pre-existing nanoparticles for loading additional payloads. Aurum nanoparticles containing therapeutic agents are utilized in this process. Initially, the surface of the gold nanoparticles is functionalized with cyclodextrins. Subsequently, additional therapeutic agents are loaded into the cavities of the cyclodextrins on the nanoparticle surface. Under hypoxic conditions, the nanoparticles degrade, resulting in the release of all therapeutic agents. Created with ChemBioDraw Ultra 12.0.

In summary, cyclodextrins are primarily used as part of nanoaggregates. Two potential avenues for investigation have been identified: the self-assembly of nanoparticles based on cyclodextrins, and the functionalization of the surface of pre-existing nanoparticles with the objective of enhancing their properties. Such supramolecular systems act both as probes for hypoxia visualization and as hypoxia-targeting agents.

## Pillararenes as an Integral Component of Multifunctional Hypoxia-Sensitive Systems

Pillar[n]arenes represent a novel class of macrocyclic, pillar-shaped host compounds.^85^ In comparison to other host molecules, pillar[n]arenes have characteristic highly symmetric columnar structures that are similar in many respects to those of the highly symmetric pumpkin host cucurbit[n]urils. The repeating units of pillar[n]arenes are composed of phenolic fragments, making them structurally similar to calix[n]arenes. Furthermore, pillar[n]arenes possess numerous phenolic moieties on both rims, making them very similar to the highly functionalized macrocyclic cyclodextrins. From the discussion above, it is evident that pillar[n]arenes exhibit a combination of distinctive characteristics found in different macrocyclic compounds.86 Due to these properties, pillararenes have rapidly gained attention in the field of macrocyclic chemistry. Consequently, it is not surprising that columnar host molecules have also found applications as part of hypoxia-responsive systems.

In particular, a supramolecular nanoplatform activated by the tumor microenvironment based on host–guest interaction and self-assembly was established.^87^ Pillar[5]arene 13-based monomer and oligo(ethylene glycol) methyl ether methacrylate were polymerized via RAFT polymerization to fabricate an amphiphilic polymer, which serves as the multipoint host motif. The phototherapeutic agent, cyano-functionalized cyanine, and the chemotherapeutic drug, pyridinyl-functionalized camptothecin, were used as guest molecules in the resulting supramolecular polymer. The multifunctional platforms were obtained by co-assembly of the supramolecular polymer and the mitochondrial respiratory inhibitor atoquone in aqueous solution. Considering that the hypoxic microenvironment is weakly acidic, the tertiary amine-containing repeat units in polymer could be protonated, which led to the phase transition of polymer from hydrophobic to hydrophilic. Meanwhile, the nanoparticles would simultaneously dissociate and release atoquone and pyridinyl-functionalized camptothecin due to the protonation of polymer and pyridine units in pyridinyl-functionalized camptothecin (**Figures 12** and **13**). Notably, in an acidic hypoxic medium, the fluorescence of the nanocatalysts obtained was significantly improved compared to a neutral medium. This allows for the implementation of anticancer therapy under bioimaging control. The proposed supramolecular platform rationally combined the functions of atoquone-boosted PDT, PTT and chemotherapy, resulting in the complementary advantage of each single therapy and better therapeutic efficacy. This study has been conducted to evaluate the efficacy of the synergistic trimodal combination therapy in inhibiting tumors. The systems demonstrated high therapeutic efficacy against 4T1 cancer cells. Additionally, the antitumor effect of supramolecular nanoparticles was evaluated in nude mice bearing 4T1 tumors. The research findings indicated that the nanoparticles exhibited low biotoxicity toward normal tissues, while causing significant damage to the tumor tissues. Thus, this study proposes a novel strategy for the development of high-performance combination therapies, which show considerable promise for clinical antitumor treatment.

**Figure 12:**
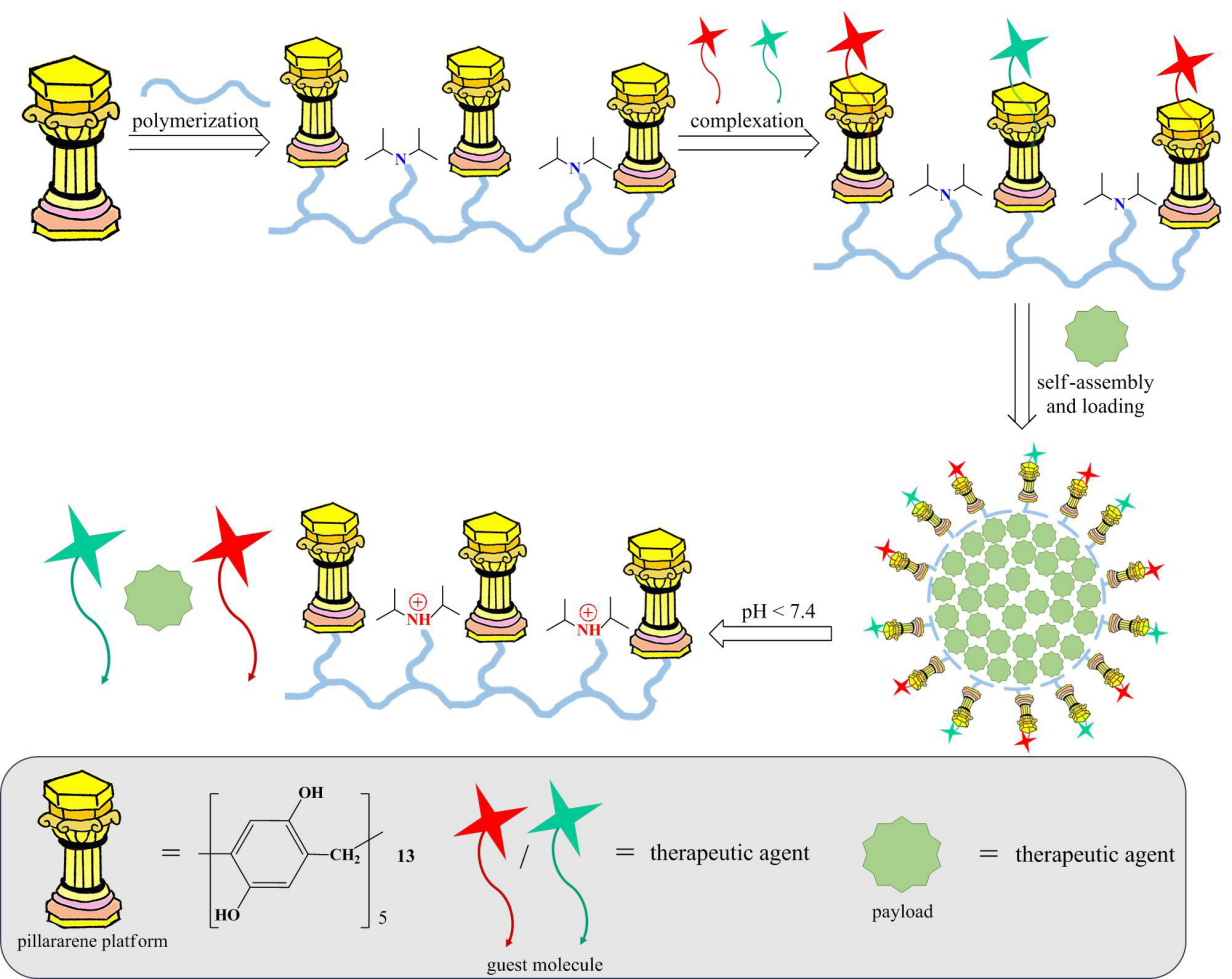
The combination cancer therapy of pillararene-based supramolecular platforms activated by the tumor microenvironment. The synthesis of a polymer based on Pillar[5]arene and oligo(ethylene glycol) methyl ether methacrylate takes place first. This is followed by the formation of a supramolecular polymer through the complexation of pillararenes with guest molecules (therapeutic agents). Next, a supramolecular nanoplatform is created via the self-assembly of the supramolecular polymer with another therapeutic agent. In an acidic hypoxic environment, the nanoplatform degrades, leading to the release of all therapeutic agents. Created with ChemBioDraw Ultra 12.0.

**Figure 13:**
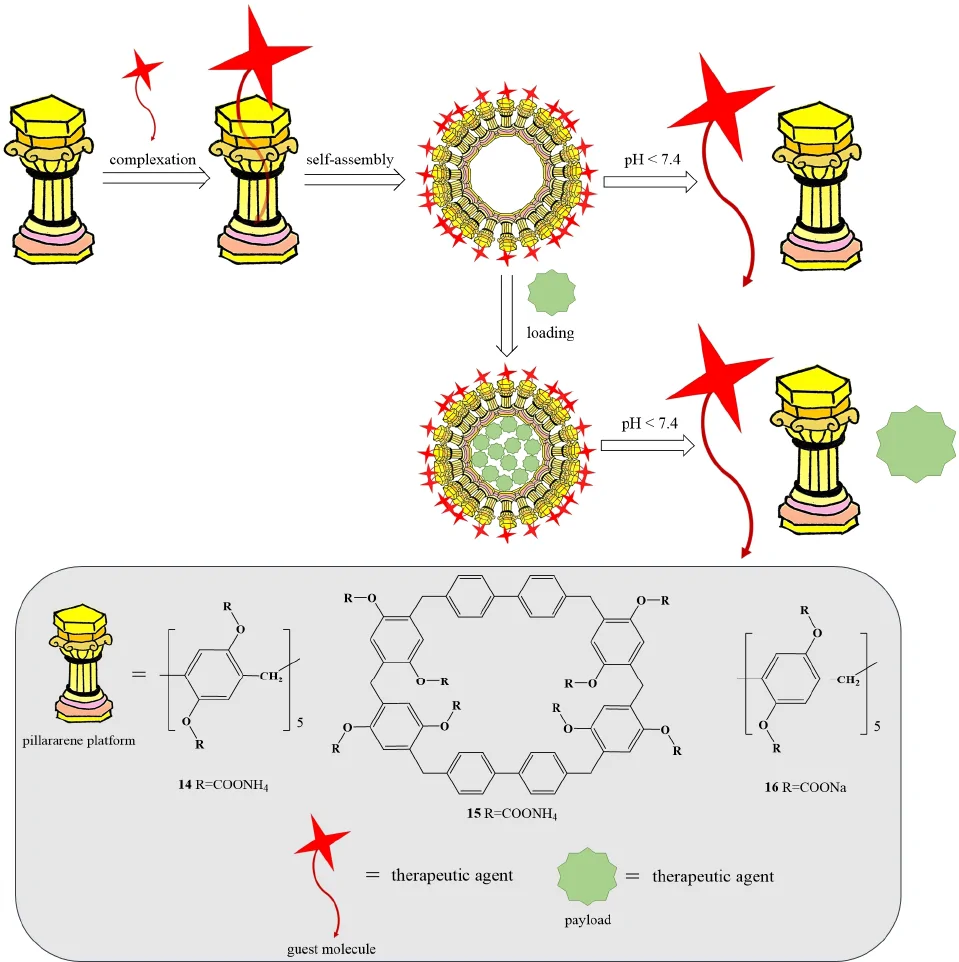
A general approach for creating hypoxia-sensing systems based on pillararenes for anticancer therapy. In the initial stage, host-guest complexes are formed using pillararene derivatives and guest molecules (therapeutic agents). The next step involves the self-assembly of these complexes into nanoparticles. In an acidic hypoxic environment, the receptor groups of the Pillar[5]arene become protonated, leading to the disintegration of the nanoparticles and the release of the guest molecules. Additionally, there is an option where the resulting nanoparticles are loaded with additional therapeutic agents. ChemBioDraw Ultra 12.0.

Another example of pillararenes presents a supramolecular systems for PDT based on a host–guest interaction between carboxylated pillar[5]arenes and an amphiphilic photosensitizer, namely functionalized L-arginine benzophenothiazine.^88^ This amphiphilic complex can self-assemble in water to form nanoparticles with negatively charged surfaces. In a hypoxic environment with a low pH, the carboxyl groups of pillar[5]arene become protonated, leading to disassembly and the release of the guest photosensitizer, which can efficiently generate oxygen under light irradiation via PDT directly damaging cancer cells. This research provides a strategy that has the potential to enhance the effectiveness of cancer therapy by generating multiple hypertoxic reactive species.

The next hypoxia-sensitive supramolecular system for PDT and chemotherapy has been developed based on tetrameric porphyrin-central poly(l-lysine-azobenzene-chlorambucil) and an anionic water-soluble [2]biphenyl-extended-pillar[6]arene. This system utilizes a synergy of hydrophobic interactions, π–π stacking, and host–guest interactions.^89^ In an acidic hypoxic environment, the self-aggregated particle disintegrates as the water-soluble salt form of the functionalized pillar[6]arene transitions to an acidic form with reduced water solubility. Additionally, in the presence of azoreductase, the azo bonds are cleaved, leading to the release of the activated drug chlorambucil. Upon laser irradiation, the central porphyrin fragment converts oxygen to its singlet state, specifically targeting and killing cancer cells. Both *in vitro* and *in vivo* results confirm the excellent antitumor efficacy of this supramolecular system, demonstrating a significant synergistic effect. This study also presents a promising approach for developing a potent nanoplatform for hypoxia-activated combination therapy.^90^

Theranostic supramolecular vesicles based on derivatives of pillar[5]arene and diketopyrrolopyrrol have also been proposed.^90^ The guest molecule absorbs in the near-infrared region and also serves as a photothermal agent and photosensitizer. The vesicles demonstrate a good encapsulation capacity for the anticancer drug tirapazamine. As this system uses the salt form of functionalized pillar[5]arene, these vesicles can disintegrate in an acidic hypoxic environment and release tirapazamine into tumor cells. A single irradiation with near-infrared light generates hyperthermic temperatures and singlet oxygen molecules. In addition, tirapazimine can be reduced to cytotoxic oxidative radicals in a hypoxic environment during PDT, further contributing to the synergistic enhancement of cancer therapy. Thus, the use of a supramolecular platform is a promising approach for effective tumor treatment through simultaneous combined PTT, PDT and PDT-activated hypoxia chemotherapy.

In conclusion, the pillararene-based supramolecular systems presented in the literature represent self-assembled nanoaggregates for the targeted delivery of therapeutic agents to cancer cells. The aforementioned examples employ ester derivatives of pillararenes, which are capable of protonation in an acidic hypoxic environment, resulting in the disintegration of the nanoparticles and the release of the payload. In these systems, pillararenes fulfill the function of a hypoxia-responsive element and also act as containers for therapeutic agents through host–guest interactions.

## Self-Assembly without Macrocycles

We have previously reviewed examples of hypoxia-sensitive supramolecular systems in the context of the macrocyclic host molecules employed. However, it is also worth paying attention to nanoaggregates obtained without the involvement of macrocycles. The driving force for the self-assembly of such systems is the formation of supramolecular interactions, which is a fundamental postulate of the non-covalent approach. The general strategy for obtaining such systems by self-assembly is summarized in **Figure 14**.

**Figure 14:**
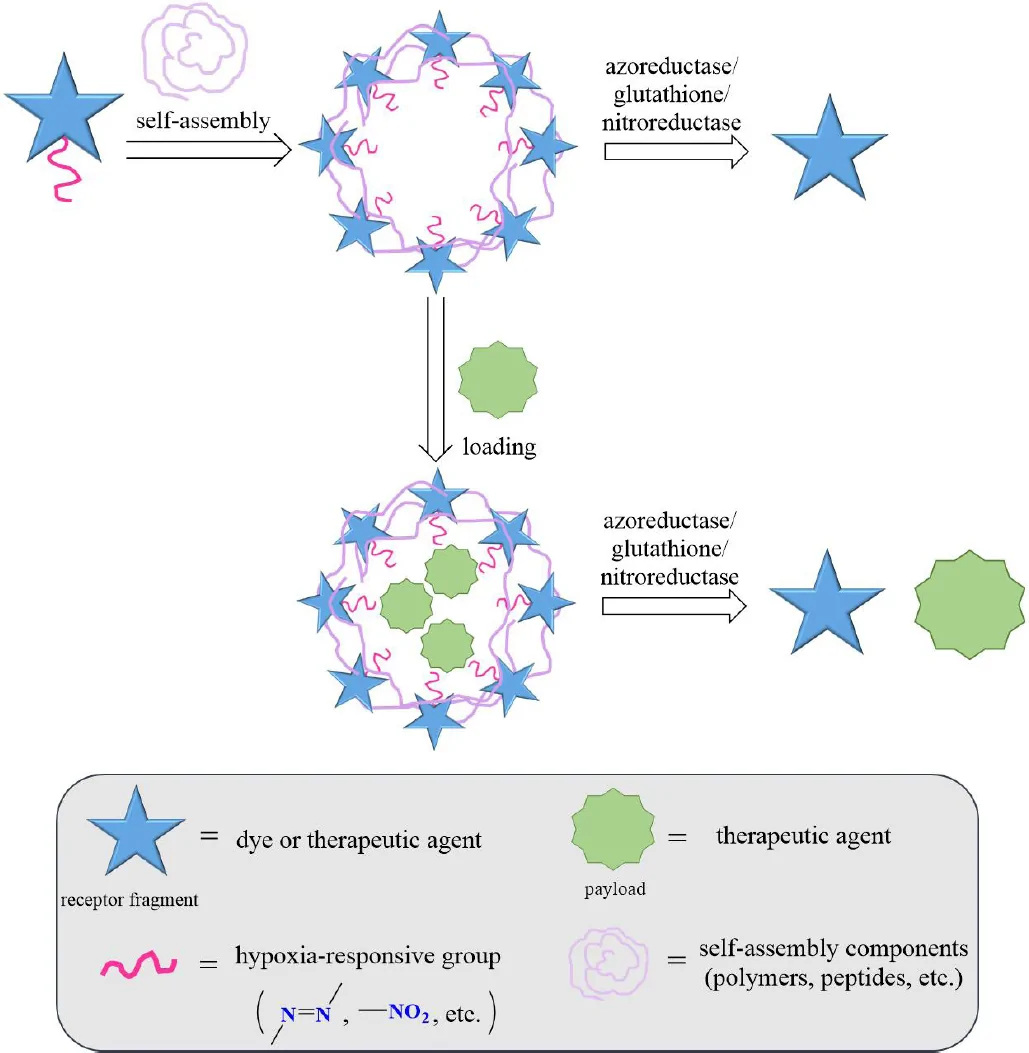
A self-assembled hypoxia-responsive system. Nanoparticles are formed using dyes or therapeutic agents that contain hypoxia-responsive groups. Under hypoxic conditions, these nanoparticles disintegrate, resulting in the release of the therapeutic agents. Additionally, there is an option where the produced nanoparticles are loaded with extra therapeutic agents. Created with ChemBioDraw Ultra 12.0.

In particular, a supramolecular complex based on a metal-organic capsule, in which the guest molecule is a nitro compound containing a phenanthro[9,10-d]imidazole group.^91^ Under the influence of nitroreductase and hypoxic conditions, the nitro group is reduced to an amino group, which leads to the appearance of luminescence from the guest molecule. The proposed signal communication method establishes a linear relationship between nitroreductase content and fluorescence intensity, with ultrafast equilibrium achieved within seconds in solution detection. Monitoring of nitroreductase in cells and mice has been demonstrated to occur at a faster rate than with traditional probes, thus showing the superiority of this approach over traditional nitroreductase imaging and early tumor diagnosis methods. Therefore, the results obtained may be useful for bioimaging and biomimetic catalysis.

The use of supramolecular vesicles loaded with a photosensitizer and therapeutic agent has been proposed as a means of efficiently eliminating hypoxic cancer cells and tumors.^92^ The process of self-assembly of amino acids protected by fluorenylmethyloxycarbonyl is initiated by Fe^3+^. In the hypoxic environment, glutathione forms a complex with Fe^3+^ within the nanovesicles, leading to the disruption of various non-covalent interactions, disassembly of the nanovesicles, and the subsequent release of the payload. The resulting nanosystems demonstrated robust stability under physiological conditions and were able to successfully down regulate hypoxia-inducible factor 1-alpha levels and alleviate hypoxia in HT29 cells via Fe^3+^-stimulated conversion of hydrogen peroxide to oxygen. This nanosystem also exhibited high PDT efficacy against HT29 cells under both normoxic and hypoxic conditions, and its photocytotoxicity was independent of the surrounding oxygen level. The addition of a therapeutic component to the nanosystems resulted in enhanced toxicity towards HT29 cells, demonstrating the synergistic effect of zinc (II) phthalocyanine and acriflavine. Furthermore, the nanosystem demonstrated accumulation in the tumor of HT29 tumor-bearing mice, likely through an enhanced permeability and retention effect. Irradiation of the nanosystems with light led to significant inhibition of tumor growth in nude mice and attenuation of tumor hypoxia. These meticulously designed Fe^3+^-driven self-organizing nanosystems are, thus, highly effective and versatile photosensitizing agents that can address the hypoxia of PDT. This work also presents a novel conceptual design of supramolecular therapeutic nanozymes constructed from simple biomolecules and metal ions, offering a promising and versatile way to ablate hypoxic tumors.

A supramolecular nanoprobe was proposed, comprising the loading of azobenzene into nanoparticles formed by an amphiphilic polymer. The polymer contains highly emissive tetraphenylethylene fluorophores in an aggregated state.^93^ Following the introduction of azobenzene, the fluorescence emission of the nanoparticle was extinguished. However, under hypoxia, azobenzene decomposes due to the action of azo reductase, thereby restoring the fluorescence of the nanoprobe. The proposed system allows for the visualization of hypoxia, which was successfully demonstrated on A549 cells cultured in an oxygen-deficient medium.

Furthermore, supramolecular peptide probes based on the amino acid 2-nitroimidazol-1-yl alanine were prepared.^94^ The synthesized tripeptide was also functionalized with the fluorescent dye IR780, with the 2-nitroimidazole fragment acting as an IR780 fluorescence quencher. Under hypoxic conditions, the nitroreductase enzyme converts the nitroimidazole group to an aminoimidazole group, thereby restoring the fluorescence intensity of the IR780 fragment present in the peptide. The efficacy of the obtained systems was evaluated in vivo using murine models. The results demonstrated that the supramolecular probes accumulated primarily in tumor sites and penetrated solid tumors that had developed as a consequence of morphological transformation. This allowed for the effective fluorescence imaging of tumor cells in hypoxic areas.

Other examples of supramolecular self-assembly serve as means of delivering anticancer drugs to cancer cells. In particular, vesicles based on an azo-naphthylamide derivative undergo disruption under hypoxic conditions, which causes the release of the loaded curcumin.^95^ The efficacy of the obtained systems was demonstrated in B16F10 cells (cancer cells) in a hypoxic environment. Another example is that of polymeric micelles loaded with DOX.^96^ The micelle shell contains 2-nitroimidazole fragments, which permit the micelles to disintegrate by nitroreductase in hypoxic cells, thereby facilitating the release of DOX. The anticancer efficacy of DOX-loaded micelles was evaluated in MCF-7 human breast adenocarcinoma cells. Nanostructures obtained by self-assembly of an amphiphilic polymer are proposed as a supramolecular system for chemo-PDT.^97^ The supramolecular polymer has been designed on the basis of ruthenium complexes in which terpyridine ligands are modified with azo bonds. In the hypoxic microenvironment of tumor hypoxia, the polymer undergoes selective activation and degradation under the action of azo reductase, leading to the release of the ruthenium complexes. Notably, the subsequent application of red light irradiation exacerbates the hypoxia and enhances the release of ruthenium complexes.

The aforementioned examples of supramolecular self-assembly encompass probes that are responsive to azoreductase, nitroreductase, and glutathione. These enzymes are typical targets for hypoxia-sensitive sensors and prodrugs. Therefore, the approach to sensor design presented in this section is analogous to that of the previously discussed supramolecular probes involving macrocyclic molecules. Nevertheless, the absence of supplementary macrocyclic cavities limits the potential for loading diverse variants of therapeutic agents. In comparison to the nanoaggregates discussed in other sections, there is a paucity of systems for combined anticancer therapy.

In this review, we focused on supramolecular systems for visualization hypoxia, specifically based on several classes of macrocycles: (thia)calixarenes, cyclodextrins, pillararenes, and cucurbiturils. Despite the numerous advantages of smart, self-organizing supramolecular systems over covalent methods for detecting hypoxia, several drawbacks must be considered. These include a relatively low specificity in identifying hypoxic regions when using targets like reductive enzymes, a reduction in water solubility due to modifications in the macrocyclic structure, and the potential for adverse effects when applied to cells with various modified macrocyclic systems and their complexes.

## Conclusion

Therefore, the supramolecular methodology for the fabrication of sensors and prodrugs responsive to hypoxia has been trialed on different classes of macrocyclic compounds. Moreover, this concept has been employed for the synthesis of nanoaggregates without the involvement of macrocycles. **Table 1** summarizes the main results of the analysis of the considered supramolecular systems. **Table 1** suggests that within a single class of macrocycles, strategies for responding to a particular hypoxia target are often realized. It is therefore expected that the next stage of research will be to develop macrocyclic derivatives with other functional groups that respond to other targets. An important point is also that some of the therapeutic agents employed exhibit poor solubility. Nevertheless, this limitation is eliminated when the drugs are encapsulated in the cavities of macrocyclic compounds, thereby significantly expanding the scope of their applicability. Among the guest molecules under investigation, those activated by hypoxia warrant particular attention. For example, the bioreductive prodrug AQ4N, which exhibits combined therapeutic effects, is frequently employed in the aforementioned supramolecular strategies. The key finding is that hypoxia imaging systems have been successfully modified into drug delivery systems for various types of anticancer therapies, including combination therapies. This variability is easily achieved through non-covalent assembly, again demonstrating the advantage of the supramolecular approach over the covalent strategy.

**Table 1:** A classification of proposed hypoxia-responsive systems is presented based on a supramolecular approach

Macrocycle	Guest molecule	Hypoxia target	Application
Calix[n]arene (*n* = 4, 5)/thiacalix[4]arene	Dye;	Azoreductase;	Discrete complexes;
	Photodynamic agent;	Glutathione	Hydrogel;
	Photothermal agent;		Nanoparticles
	Hypoxia-activated agent;		
	Chemotherapeutic drug		
Cucurbit[n]uril (*n* = 6–7)	Hypoxia-activated agent;	pH < 7.4;	Discrete complexes;
	Photothermal agent;	Azoreductase;	Nanoparticles
	Chemotherapeutic drug	Reductive enzymes	
Cyclodextrin (*n* = 6–7)	Dye;	pH < 7.4;	Nanoparticles
	Chemotherapeutic drug;	Azoreductase;	
	Hypoxia-activated agent;	Glutathione;	
	Enzymatic agent;	Nitroreductase	
	Photothermal agent		
Pillar[n]arene (*n* = 5–6)	Photodynamic agent;	pH < 7.4	Nanoparticles
	Photothermal agent;		
	Chemotherapeutic drug		
Self-assembly with non-macrocyclic components (polymers, peptides, etc.)	Dye;	Nitroreductase;	Nanoparticles
	Photodynamic agent;	Glutathione;	
	Chemotherapeutic drug	Azoreductase	

In summary, the presented review demonstrates the significant potential of supramolecular hypoxia-sensitive systems for the diagnosis and treatment of cancer. The synthesis of various macrocyclic derivatives and their subsequent self-assembly into nanoaggregates has enabled extensive study and evaluation of the response processes to a range of hypoxic stimuli. It is important to note that the non-covalent approach discussed is relatively recent, and many of the proposed strategies have been presented as single examples only. Nevertheless, it seems reasonable to suggest that the selected studies will continue to make a valuable contribution to this field in the future. The developed concepts of supramolecular hypoxia-responsive systems have already been successfully demonstrated in living subjects, allowing not only for the diagnosis of hypoxic cancer cells but also their selective killing. Furthermore, the combination of different therapeutic agents in a single supramolecular ensemble allows for the simultaneous implementation of multiple imaging-guided cancer therapies. These advances not only contribute to the advancement of our fundamental knowledge, but also offer practical solutions to world health problems. The development of innovative supramolecular systems represents a significant advancement in the transition towards personalized medicine, theranostics, and high-performance healthcare technologies. It is important to note that the use of supramolecular systems that respond to hypoxic tumor microenvironments is a novel approach, and clinical trials for these systems have not yet been carried out. It can be anticipated that several challenges may arise on the path to clinical application of supramolecular systems, including potential side effects, high costs, research complexities, and the risk of failure.

**Facts**

1. Non-covalent supramolecular design strategies offer a promising alternative for developing hypoxia-sensitive sensors and prodrugs, bypassing the complex syntheses of covalent approaches by relying on the self-assembly of components.
2. Macrocyclic compounds (e.g. cyclodextrins, cucurbiturils and pillararenes) act as core platforms for constructing hypoxia-targeted/activated probes thanks to their molecular recognition properties. They can act as “molecular containers” for drugs and fluorescent dyes.
3. The formation of supramolecular host-guest complexes can significantly enhance the photostability of dyes, improve the signal-to-noise ratio, increase the water solubility of hydrophobic guests, reduce cytotoxicity and promote the targeted accumulation of probes in tumor cells.

**Open Questions**

1. What are the primary barriers to the clinical translation of most supramolecular hypoxia probes currently in the laboratory stage? How can their *in vivo* biosafety, metabolic pathways and long-term toxicity be systematically evaluated?
2. How can more effective macrocyclic hosts be designed and selected more effectively? The mechanisms by which different macrocyclic structures influence hypoxia response sensitivity, selectivity and biodistribution (e.g., cyclodextrins *vs*. cucurbiturils) remain unclear.
3. Could complex tumor microenvironment factors (e.g., pH, enzymes, redox status) interfere with the hypoxia-specific response of supramolecular probes? How can multi-responsive or self-calibrating intelligent systems be constructed to improve detection accuracy?

## Data Availability

*Not applicable.*
